# Isolated bilateral external iliac artery dissections with emotional stress

**DOI:** 10.1002/ccr3.3762

**Published:** 2021-01-08

**Authors:** Shun Kondo, Hiroyuki Osanai, Yusuke Sakamoto, Hiroto Uno, Kensuke Tagahara, Hirotaka Hosono, Shun Miyamoto, Shotaro Hiramatsu, Hikari Matsumoto, Teruhiro Sakaguchi, Takahiro Kanbara, Yoshihito Nakashima, Hiroshi Asano, Masayoshi Ajioka

**Affiliations:** ^1^ Department of Cardiology Tosei General Hospital Seto Japan

**Keywords:** emotional stress, external iliac artery dissection

## Abstract

We present a patient with isolated bilateral external iliac artery dissections associated with emotional stress. The diagnosis should be kept in mind in young, fit patients presenting lower back pain occurring subsequent to emotional stress.

## Graphical abstract

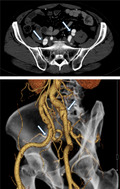

## 1 INTRODUCTION

Spontaneous and isolated bilateral external iliac artery dissection is extremely rare. We present a case of isolated bilateral external iliac artery dissection secondary to emotional stress. We believe this to be the first case with this condition secondary to emotional stress in patient without connective tissue disorder.

Spontaneous and isolated bilateral external iliac artery dissection without involvement of the aorta is extremely rare.^1^ Only a few cases regarding this condition have been previously reported. The etiologic factors of external iliac artery dissection are trauma, connective tissue disorder, atherosclerosis, and physical strain.^2^

Most of the reports presented in the literature are associated with connective tissue disorder or intense exercise. Here, we present the case of a patient with isolated bilateral external iliac artery dissections in a 50‐year‐old man with emotional stress managed by conservative treatments.

## 2 CASE HISTORY

**FIGURE 1:**
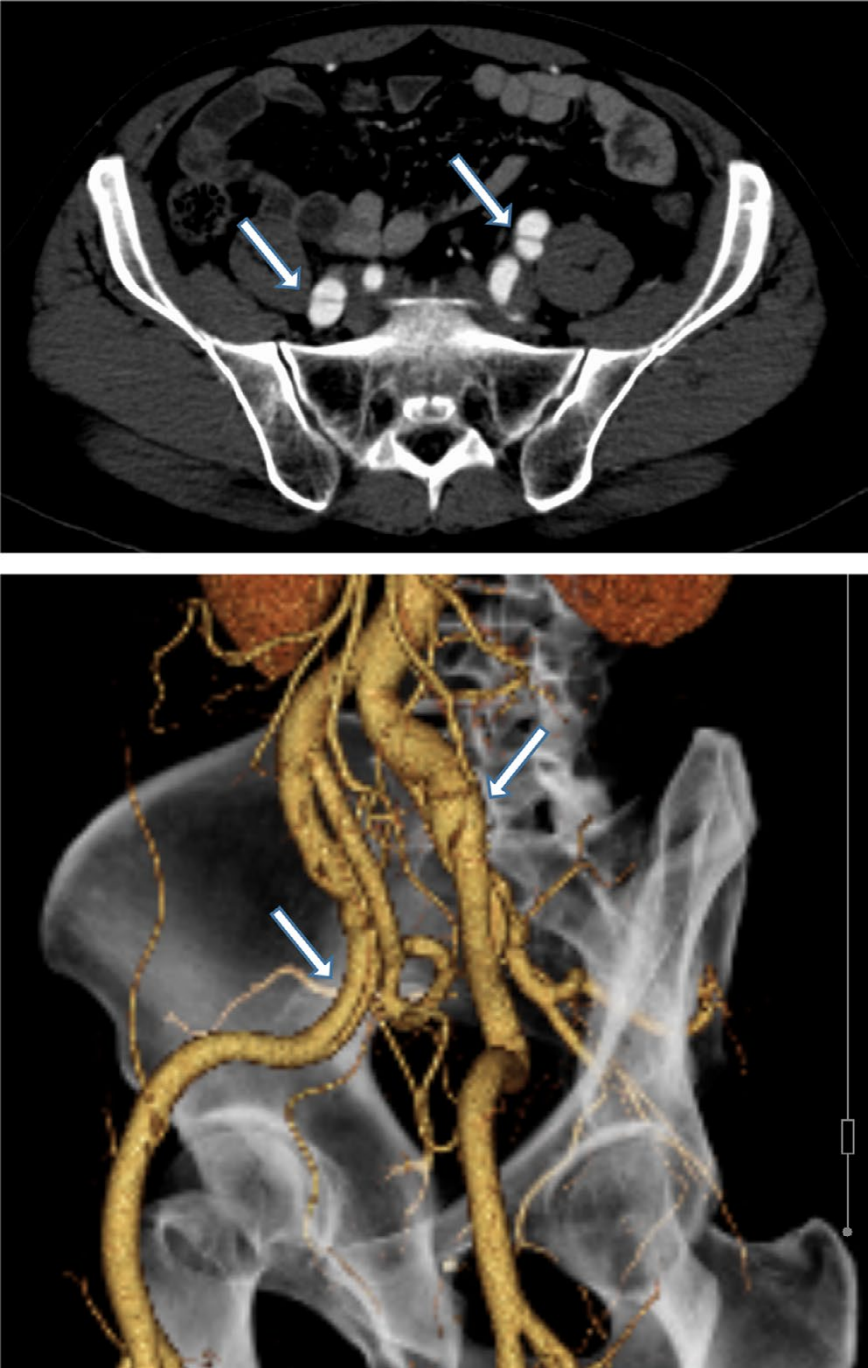
Computed tomography demonstrating bilateral external iliac artery dissection (arrows)

A 50‐year‐old man was admitted to our medical center for new‐onset lower back pain during his mother's funeral. He had no significant past medical or surgical history. He had smoked one packs‐per‐day of cigarettes for 20 years but quit 10 year ago and had no other cardiovascular risk factors. He was not prescribed any medications. There was no familial history of aortic disease, sudden death, or structural cardiac abnormalities. He denied any intense exercise. His mother died 7 days before the onset of symptoms, and he started having symptoms during her funeral. On physical examination, he was hemodynamically stable with a normal sinus rhythm of 81 beats per minute and blood pressure of 172/106 mm Hg, and femoral and distal pulses were palpable and strong bilaterally. A contrast‐enhanced computed tomography (CT) showed isolated bilateral external iliac artery dissections (Figure 1). There are no stenosis, aneurysms, or other dissections of the arterial system in ether leg. The patient's white blood cell count was 8.1 × 10^3^/μL, d‐dimer concentration was 0.27 μg/dL, low‐density lipoprotein cholesterol levels were 121 mg/dL, and HbA1c was 5.9%. An ankle‐brachial index (ABI) was 1.21 on the right side and 1.18 on the left side. The patient had no symptoms of claudication before or after hospital admission, and lower back pain was reduced by analgesic; thus, we chose conservative treatments, including antihypertensive therapy and close follow‐up. A repeated CT 4 days following symptomatic onset showed that dissections and vessel diameter were both stable. A rehabilitation program based on type B aortic dissection was undertaken. During the rehabilitation program, he had neither symptom nor claudication. He was discharged from the hospital on day 13. A follow‐up CT 1 month later showed no evidence of dissection extension. Over the course of 2 years follow‐up, the patient remained completely asymptomatic with good distal pulses, and a follow‐up CT showed no extension of the dissection. Outpatient follow‐up has been adjusted to be done every 6 months.

## 3 DISCUSSION

Spontaneous and isolated external iliac artery dissection is a rare phenomenon, especially, if there is bilateral involvement.^3^ The etiologic factors of external iliac artery dissection are trauma, connective tissue disorder, atherosclerosis, and physical strain.^2^ Connective tissue disorder includes Marfan syndrome, α1‐antitrypsin deficiency, fibromuscular dysplasia, and Ehlers‐Danlos syndrome.^4^ There have been a few reports of external iliac artery dissection in athletes or workers; cyclists are the most common, and cases in runners, golf swing trainers, and blue collar workers have also been reported.^1^

In this case, the patient had no history of trauma or excessive training, and no signs or symptoms suggestive of connective tissue disorder. Apart from past smoking history, we speculate that our patient could have been affected by his mother's death and funeral, possibly due to emotional stress. The potential impact of acute emotional stress on cardiovascular disease has frequently been assessed by evaluating the aftermath of happenstance natural disasters and adverse emotional events.^5^ For example, the etiology of Takotsubo cardiomyopathy, also known as stress‐induced cardiomyopathy or broken heart syndrome, potentially involves a hyperadrenergic state in response to acute emotional stress.^6^ Similarly, an association has been reported between acute emotional stress and acute aortic dissection.^7^ Recent studies have shown that emotional stress triggers increased sympathetic nervous systems which activate the adrenal medulla and the peripheral sympathetic nerves, leading to increased circulating levels of catecholamines (mainly adrenaline and noradrenaline, respectively), which increase blood pressure and heart rate with the effect that wall stress exceeds the tensile limit of aortic tissue, resulting in an acute aortic dissection.^5^, ^7^ There has been a report of external iliac artery dissection in a patient with Ehlers‐Danlos syndrome due to emotional stress.^8^ Similarly, emotional stress could have been a contributing factor in our patient. To the best of our knowledge, our patient represents the first reported case of acute emotional stress triggering isolated bilateral external iliac artery dissection without genetic syndrome or connective tissue disorder.

Many investigative modalities for assessing external iliac artery dissection have been proposed. An ABI has high specificity but relatively low sensitivity.^9^ An arterial duplex examination detects both stenosis and elongation of iliac arteries reliably but is user‐dependent.^10^ A CT or magnetic resonance angiography is highly sensitive in detecting stenosis, dissection, or arterial wall thickening.^9^ Management options for external iliac artery dissection are conservative treatment, open surgery, and endovascular treatment. Criteria for the treatment of patients with external iliac artery dissection are not yet defined, owing to the rarity of the condition.^11^ Conservative treatments, which include close observation, follow‐up imaging, and best medical therapies, can be suitable for patients without symptoms. In patients with asymptomatic isolated iliac artery dissection, conservative treatment was reported to be a safe option.^12^ Open surgery can be suitable for patients with a strong desire to continue their sporting lifestyle.^1^, ^9^ Endovascular treatment has an advantage over open surgery in that it is less invasive, potentially making it preferable to athletes.^1^

## 4 CONCLUSION

Isolated bilateral external iliac artery dissections associated with emotional stress in a patient without genetic syndrome or connective tissue disorder have not been previously reported in the literature to our knowledge. The diagnosis should be kept in mind in young, fit patients presenting lower back pain occurring subsequent to emotional stress.

## CONFLICTS OF INTEREST

This report was not funded by any agencies in the public, commercial, or not‐for‐profit sectors.

## **AUTHOR** **CONTRIBUTION**

Shun Kondo: conceptualized and designed the study, and involved in acquisition of data, analysis and interpretation of data, and drafting the manuscript. Hiroyuki Osanai: conceptualized and designed the study. Yusuke Sakamoto, Hiroto Uno, Kensuke Tagahara, Hirotaka Hosono, Shun Miyamoto, Shotaro Hiramatsu, Hikari Matsumoto, Teruhiro Sakaguchi, Takahiro Kanbara, Yoshihito Nakashima, Hiroshi Asano, and Masayoshi Ajioka: involved in technical help, writing, and editing assistance.

## Data Availability

All data and material collected during this study are available from the corresponding author upon reasonable request.
